# Natural Killer Cells Promote Long-Term Hepatobiliary Inflammation in a Low-Dose Rotavirus Model of Experimental Biliary Atresia

**DOI:** 10.1371/journal.pone.0127191

**Published:** 2015-05-19

**Authors:** James E. Squires, Pranavkumar Shivakumar, Reena Mourya, Kazuhiko Bessho, Stephanie Walters, Jorge A. Bezerra

**Affiliations:** 1 Department of Pediatrics of the University of Cincinnati College of Medicine, the Division of Gastroenterology, Hepatology and Nutrition and the Pediatric Liver Care Center of Cincinnati Children’s Hospital Medical Center; Cincinnati, Ohio, United States of America; 2 Division of Pediatrics, Osaka University, Osaka, Japan; Texas A&M Health Science Center, UNITED STATES

## Abstract

Biliary atresia is a rapidly progressive obstructive cholangiopathy of infants. Mechanistic studies in the mouse model of Rhesus rotavirus (RRV)-induced biliary atresia have linked the importance of effector lymphocytes to the pathogenesis of extrahepatic bile duct (EHBD) injury and obstruction in experimental biliary atresia; however, studies of the progressive liver injury have been limited by early death of newborn mice. Here, we aimed to determine 1) if a lower inoculum of RRV induces obstruction of EHBDs while allowing for ongoing liver inflammation, and 2) if NK cells regulate intrahepatic injury. The administration of 0.25x10^6^ fluorescence forming units of RRV induced an obstructive extrahepatic cholangiopathy, but allowed for restoration of the duct epithelium, increased survival, and the development of a progressive intrahepatic inflammatory injury with molecular and cellular signatures equivalent to the traditional infectious model. Investigating the mechanisms of liver injury, we found that NK cell depletion at the onset of jaundice decreased liver inflammation, suppressed the expression of fibrosis and inflammation/immunity genes, lowered plasma ALT and bilirubin and improved survival.

**Conclusions:**

Lower inoculation of RRV-induced progressive liver injury and fibrosis via NK cells. These findings point to the potential use of NK cell-depleting strategies to block progression of liver disease in biliary atresia.

## Introduction

Biliary atresia is a rapidly progressive cholangiopathy of infancy resulting from an inflammatory and fibrosing obstruction of extra- and intrahepatic bile ducts. Studies in an experimental model of biliary atresia have identified molecular and cellular mechanisms driving the pathogenesis of extrahepatic epithelial damage and duct obstruction [1–7]. Among them, natural killer (NK) cells have been demonstrated to populate the diseased livers in biliary atresia [8–12] and induce cholangiocyte injury and disruption of the extrahepatic duct epithelium in experimental atresia [13]. However, the translation of these discoveries into improved therapeutic interventions and novel treatment modalities has been hampered by the limitations of the current experimental model and the challenges in performing clinical trials in young infants with biliary atresia [14].

Many of the histological and biochemical features of extrahepatic biliary atresia can be recapitulated in newborn Balb/c mice inoculated with 1.5x10^6^ fluorescence forming units (ffu) of *Rhesus* rotavirus (RRV) [15]. This model has enabled mechanistic studies and uncovered molecular, cellular and gene-expression patterns that define experimental biliary atresia, and identified key initiating and progression events that result in extrahepatic biliary obstruction [6, 13, 16–20]. However, the severity of injury and the rapid progression of the disease in affected mice result in universal early mortality. As a consequence, the model has not been suitable for studies of the pathogenesis of the intrahepatic disease, which largely dictates the outcome in human patients after Kasai hepatoportoenterostomy (HPE).

Here we aimed at investigating the mechanisms of liver injury in biliary atresia. To this end, we modified the current model of experimental biliary atresia by lowering the infectious dose of RRV, which produced an intrahepatic cholangiopathy with prolonged survival and ongoing liver injury beyond 2 weeks of life. In these mice, depletion of NK cells at the onset of jaundice improved obstructive sequela and prevented the progression of intrahepatic hepatobiliary injury through a dampening of the intrahepatic immune activated and inflammatory responses.

## Materials and Methods

### Infection of neonatal mice with RRV

Balb/c mice were maintained in a specific pathogen-free vivarium and housed in a room with a 12-hour dark-light cycle. Throughout all experiments, mice were monitored daily to assure appropriate humane conditions and comprehensive measures were taken to minimize animal suffering in consultation with institutional veterinary services. Newborn Balb/c mice were injected with 0.9% saline solution (controls) or 1.5x10^6^ ffu RRV with phenotyping obtained by measurement of daily weights and monitoring for the development of jaundice of the non-fur covered skin and survival as described previously [4]. Additional groups were generated by injections with lower doses of RRV at 1.0x10^6^, 0.75x10^6^ or 0.25x10^6^ ffu, followed by phenotyping as above. With regards to survival studies, mice were monitored daily for the development of cholestasis and survival. If mice became moribund or expressed clinical or behavioral signs of inactivity, self-mutilation, cannibalism by littermates or lack of responsiveness, they were euthanized following the principles of humane endpoints (in stead of experimental endpoints). No analgesics or anesthetics were used to minimize suffering in order to keep animals in the experimental design. When indicated as discussed above, animal sacrifice was performed by CO_2_ asphyxiation followed by blood exsanguination via cutting of the inferior vena cava. Additionally, mice were sacrificed at 7, 14 or 21 days after saline or RRV injection. Animal sacrifice was performed by CO_2_ asphyxiation followed by blood exsanguination via cutting of the inferior vena cava. At these time points, gross appearance of the livers and bile ducts were recorded and livers were harvested for RNA isolation, histological analyses and for isolation of mononuclear cells. This study was carried out in strict accordance with the recommendations in the Guide for the Care and Use of Laboratory Animals of the National Institutes of Health. The Institutional Animal Care and Use Committee of the Children’s Hospital Research Foundation (Cincinnati, Ohio, USA) approved all animal protocols (Protocol Number: 2D04029).

### Colorimetric assays

At the time of sacrifice, blood was obtained by cardiac puncture and plasma was separated by centrifugation at 1200 rpm x12 min at 4°C. Plasma ALT and total bilirubin concentrations were measured with DiscretPak ALT Reagent Kit (Catachem, Bridgeport, CT) and Total Bilirubin Reagent Set (Pointe Scientific Inc., Canton, MI), respectively according to the manufacturers’ instructions. Photometric absorbance for both assays was read on a Synergy H1 Hybrid Reader (BioTek, Winooski, VT) at 555 nm.

### Plasma cytokine assays

Mouse plasma was collected by centrifugation of heparinized blood at 5000 rpm for 10 minutes. Levels of Tnfa, IL-1b, and IL-6, were determined by Milliplex kits (Millipore, Billerica, MA) according to the manufacturer’s protocol. Briefly, in a 96 well multiscreen filter plate, 25μL sample was incubated with 25μL antibody coated beads overnight at 4°C, followed by incubation with 25μL of secondary antibody for 1 hour at room temperature. Finally, 25μL of streptavidin-RPE was added for 30 minutes, and then read using the Bio-Plex (Bio-Rad, Hercules, CA).

### Histopathology and immunofluorescence staining

Livers and extrahepatic bile ducts from experimental mice were micro-dissected using a stereomicroscope. Tissues were paraffin-embedded, sectioned and stained with H&E, Masson’s Trichrome and Sirius Red depending on experimental design. Microtome sections were submitted to blocking of nonspecific binding by using normal donkey serum; and incubated with rabbit anti-cow cytokeratin antibody (Dako North America Inc., Carpinteria, California, USA) to stain cholangiocytes. Specific signals were detected using FITC-conjugated donkey anti-rabbit conjugated secondary antibodies (Jackson ImmunoResearch Laboratories Inc., West Grove, Pennsylvania, USA). Images were captured using Olympus BX51 microscope (Olympus America Inc., Center Valley, Pennsylvania, USA) and cellSens Dimension digital imaging software (Olympus corporation, Version 1.8.1).

### Gene expression analysis

RNA was isolated from livers of RRV- or saline-injected mice using RNeasy Mini Kit, according to the manufacturer’s protocol (Qiagen, Valencia, California, USA). RNA integrity was confirmed by agarose gel electrophoresis as described previously [21]. All samples were reverse transcribed and used in real-time quantitative PCR (qPCR). Primer sequences and qPCR cycling parameters to quantify expression of murine *Ifng*, *Cxcl9*, *Cxcl10*, *Igj*, *Ltf*, *Mmp7*, *Lcn2*, *S100a8*, *S100a9*, *Sma*, *Timp1*, *Mmp8*, *Mmp9*, *Col1a1*, *Ncr1*, *Klrk1*, *Klrd1*, *Klrc1*, *FasL and Tnfa* are listed in S1 Table.

### Flow cytometric analysis

Mononuclear cells were isolated from freshly harvested livers of RRV- or saline-injected mice by homogenization with a gentleMACS dissociator (Miltenyi Biotec, San Diego, California, USA), sifted through a 40-μm cell strainer, spun at 2000 rotations per minute using a Eppendorf 5430R centrifuge (Eppendorf, Hauppauge, New York, USA) at 4°C, and underwent red blood cell lysis as described previously [4, 6]. Cellular phenotyping was determined by the detection of surface markers as previously described [4, 6] using the following flourochrome-conjugated, species-specific monoclonal antibodies: FITC anti-mouse CD3 (17A2, IgG_2b_) and allophyocyanin (APC) conjugated anti-NK (CD49b; DX5, IgM) purchased from eBioscience (San Diego, California, USA); Pacific Blue-anti-mouse CD4 (RM4-5, IgG_2b_) from BioLegend (San Diego, California, USA) and PerCP-Cy5.5 anti-mouse CD8 (ly2.2, 53–6.7) from Leinco Technologies (St. Louis, Missouri, USA). Stained cells were analyzed in a FACSCalibur dual laser flow-cytometer (BD Bioscience, San Jose, California, USA) and data were analyzed using FlowJo software (Tree Star Inc., Ashland, Oregon, USA) with cell populations selected as described previously [13].

### Microarray analyses

Genome-wide expression datasets for extrahepatic bile ducts were generated and reported by us previously (deposited in GEO:GSE46995) [22]. Datasets were analyzed using GeneSpring GX11.5 platform, beginning with a selection of genes whose expression differed by ≥ 2-fold among groups of extrahepatic bile ducts from mice that were infected with 1.5x10^6^ ffu RRV and normal saline controls according to unpaired t-test with a significance of < 0.05 and Benjamin-Hochberg multiple testing correction (false discovery rate of < 0.05). ToppFun algorithm of Toppgene Suite (http://toppgene.cchmc.org/) was utilized to identify gene function, and CIMminer to depict gene ontology/biological relatedness [22].

### Statistical analysis

Results from flow cytometric analyses and real-time PCR were performed using Prism 5.0c (GraphPad Software, San Diego, California, USA). Values were expressed as mean ± standard deviation and statistical significance was determined by two-tailed unpaired t-test with 95% confidence interval and a significance level of p<0.05. Kaplan Meier analysis was used to assess the difference in survival rates.

## Results

### Low doses of RRV induce extrahepatic cholangiopathy and progressive intrahepatic hepatobiliay injury

**Fig 1:**
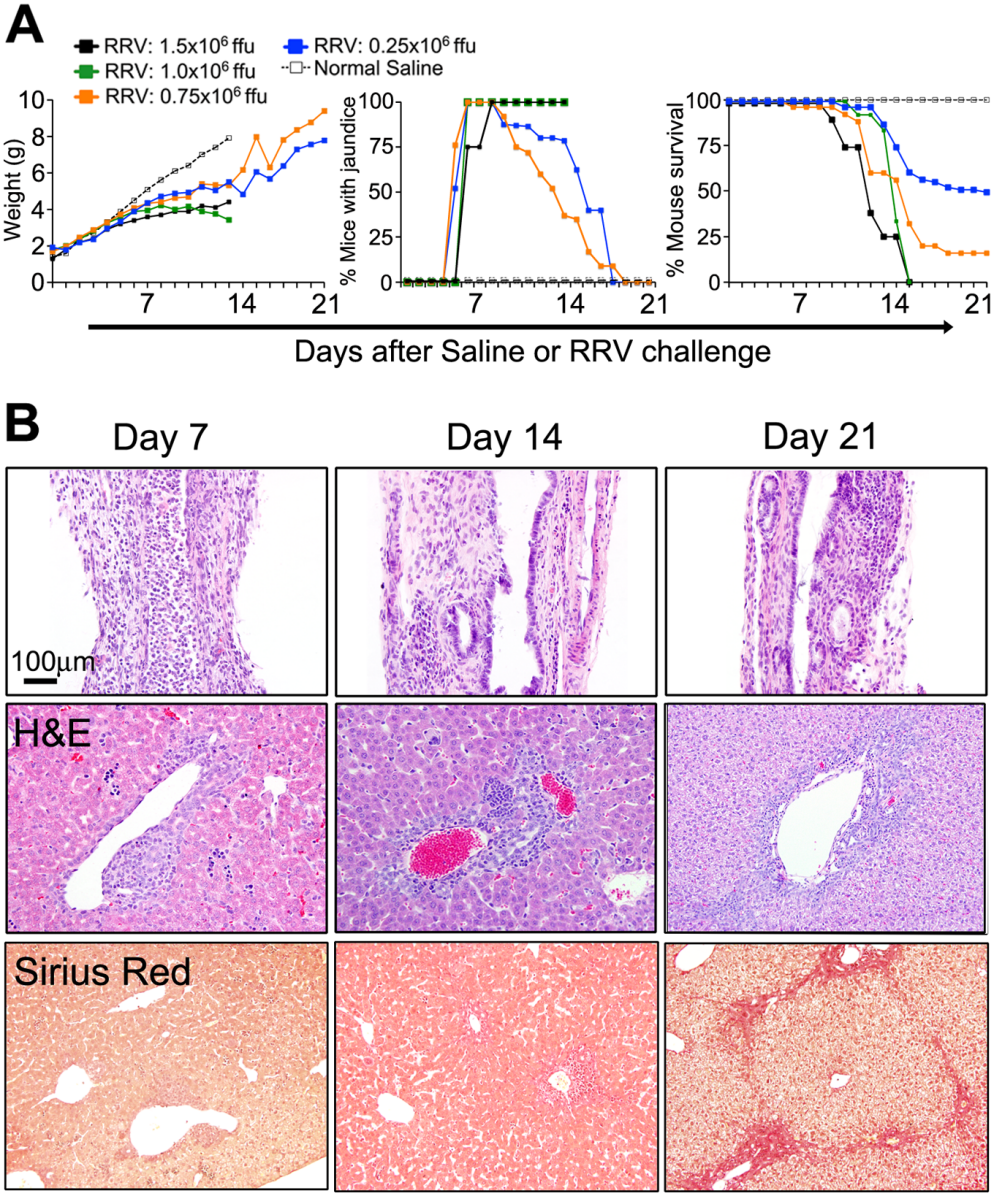
Impact of 0.25x10^6^ ffu (low-dose) RRV on disease phenotype and outcome. (A) Weight gain, jaundice and survival following different doses of RRV in newborn mice shows that 48% of mice that received the low dose survived through day of life 21 (N = 12–25 per group). (B) Sections of extrahepatic bile ducts (top panels) from low-dose challenged mice show an inflammatory obstruction of the duct lumen on day 7 with subsequent clearance by day 14 and persistent focal epithelial and submucosal injury on day 21. Accompanying liver sections (middle and lower panels) show persistent periportal inflammatory infiltrate from days 7–21, with the development of fibrosis on day 21. Tissue sections stained with H&E or Sirius Red (where noted); liver magnification x40 for day 7 and 14 H&E livers, and x20 for day 21 H&E and Sirius Red; N = 4–5 per group at each time point.

Administration of 1.5x10^6^ ffu of RRV (high-dose) in the first 24 hours of life in Balb/c mice resulted in jaundice, growth retardation and acholic stools secondary to obstruction of extrahepatic bile ducts (EHBD), as described previously [19, 20]. Universal mortality occurred in infected mice by day 15 following viral challenge. Such a rapid progression to death prevented the development of a progressive intrahepatic hepatobiliary injury. To explore the relationship between viral load, biliary obstruction and mortality, we injected varying doses of RRV into newborn mice. We found that all doses of RRV resulted in poor growth and the development of an obstructive biliary phenotype (defined by jaundice) in 100% of mice (Fig 1A). However, 0.25x10^6^ ffu (low-dose) of RRV was accompanied by an improved survival, with 48% of mice alive through day of life 21; surviving mice displayed better weight gain as well as resolution of the obstructive phenotype (Fig 1A).

To understand the anatomical basis of the improvement in the cholestatic phenotype, we performed histological analyses of the livers and EHBDs following low-dose RRV. Infected mice had a timely, but temporary luminal obstruction of EHBDs by day 7, followed by a decrease in inflammation and restoration of the epithelial lining by day 14 (Fig 1B; S1 Fig). Livers of these mice showed a lymphocytic periportal expansion at days 7 and 14, which persisted through day of life 21 and was associated with the development of fibrosis shown by Sirius Red and Trichrome staining (Fig 1B and S2 Fig). Together, these data suggested that low-dose RRV induces an obstructive cholangiopathy that is transient in EHBDs and ongoing in the liver, with portal inflammation and fibrosis in mice that survive beyond 15 days.

### Low-dose RRV induces a hepatic inflammatory response akin to high-dose

**Fig 2:**
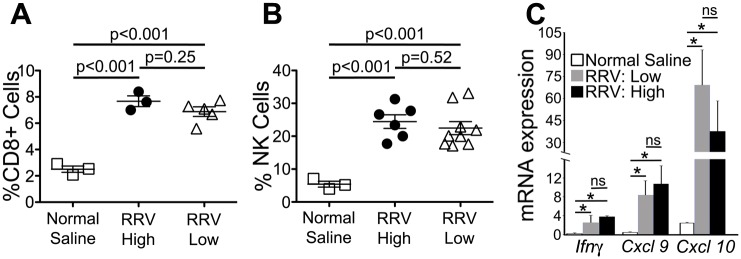
Hepatic inflammation after low-dose RRV. Flow-cytometry based quantification of hepatic mononuclear cells shows similar high populations of CD8+ T- and NK cells (A and B) and over-expression of *Ifnγ*, *Cxcl9* and *Cxcl10* (C) between low-dose (0.25x10^6^ ffu) and high-dose (1.5x10^6^ ffu) RRV (* = P<0.05, ns = not significant, N = 3–9 per group).

NK and CD8+ T-cell expansion is a well-described mechanism of cholangiocyte injury following RRV infection in experimental biliary atresia [4, 6]. To assess the appropriateness of the hepatic inflammatory response to the low-dose inoculate, we used flow cytometry-based analyses to quantify NK and CD8+ T-cell populations in livers of neonatal Balb/c mice on day 7 after intraperitoneal injection of either saline (controls), high- or low-dose RRV. We found that the CD8+ T-cell and NK cell lineages increased in the livers of both RRV infected populations compared to saline-injected controls (Fig 2A and 2B). Importantly, there was no difference between the high- and low-dose RRV infected groups. This was accompanied by a similar pattern of mRNA expression of the Th1 cytokines and chemokines *Ifnγ*, *Cxcl9* and *Cxcl10* (Fig 2C). The similar cellular and cytokine/chemokine profiles indicate that low-dose RRV induces an inflammatory response to a magnitude previously shown to initiate cholangiocyte injury and drive the development of extrahepatic obstruction in experimental biliary atresia [4, 6, 13].

### NK cell depletion at the onset of cholestasis prevents progression of liver injury after RRV

Resident hepatic NK cells are known to be key responders to infectious challenges to the liver and have been shown to initiate bile duct injury in experimental biliary atresia [13, 23, 24]. Based on these data and on reports showing that NK cells populate the portal tracts of infants with biliary atresia [11, 13], we hypothesized that NK cells play a key role in the regulation of the intrahepatic injury in experimental biliary atresia. To test this hypothesis, we depleted NK cells in newborn mice subjected to low-dose RRV at the time of duct obstruction. NK cell depletion was obtained by the administration of 30 μL of rabbit anti-asialo GM1 antiserum [known to deplete NK cells; ref [13]] starting at the onset of jaundice, typically 6 days after RRV injection, followed by daily intraperitoneal injections through day of life 14; 30 μL phosphate-buffered saline (PBS) was used as control. The administration of antiserum resulted in NK cell depletion, as determined by flow cytometry-based quantification of hepatic NK cells expressing CD49b at days of life 9 and 10 (Table 1).

**Table 1:** Efficacy of NK cell depletion following RRV challenge.

Days after RRV	% NK cells (SD)		*t* test *p* value
	PBS Control	NK Depleted	
Day 9	21.7 (4.53)	3.19 (0.33)	0.03
Day 10	20.9 (4.38)	5.25 (1.60)	0.04

Flow-cytometric quantification of hepatic NK cells after daily administration of anti-asialo GM1 antiserum or phosphate-buffered saline (PBS) beginning at the onset of jaundice (day 6 after RRV). Cells were quantified on days 9 and 10 after RRV; data expressed as average % of hepatic NK cells and standard deviation (SD).

**Fig 3:**
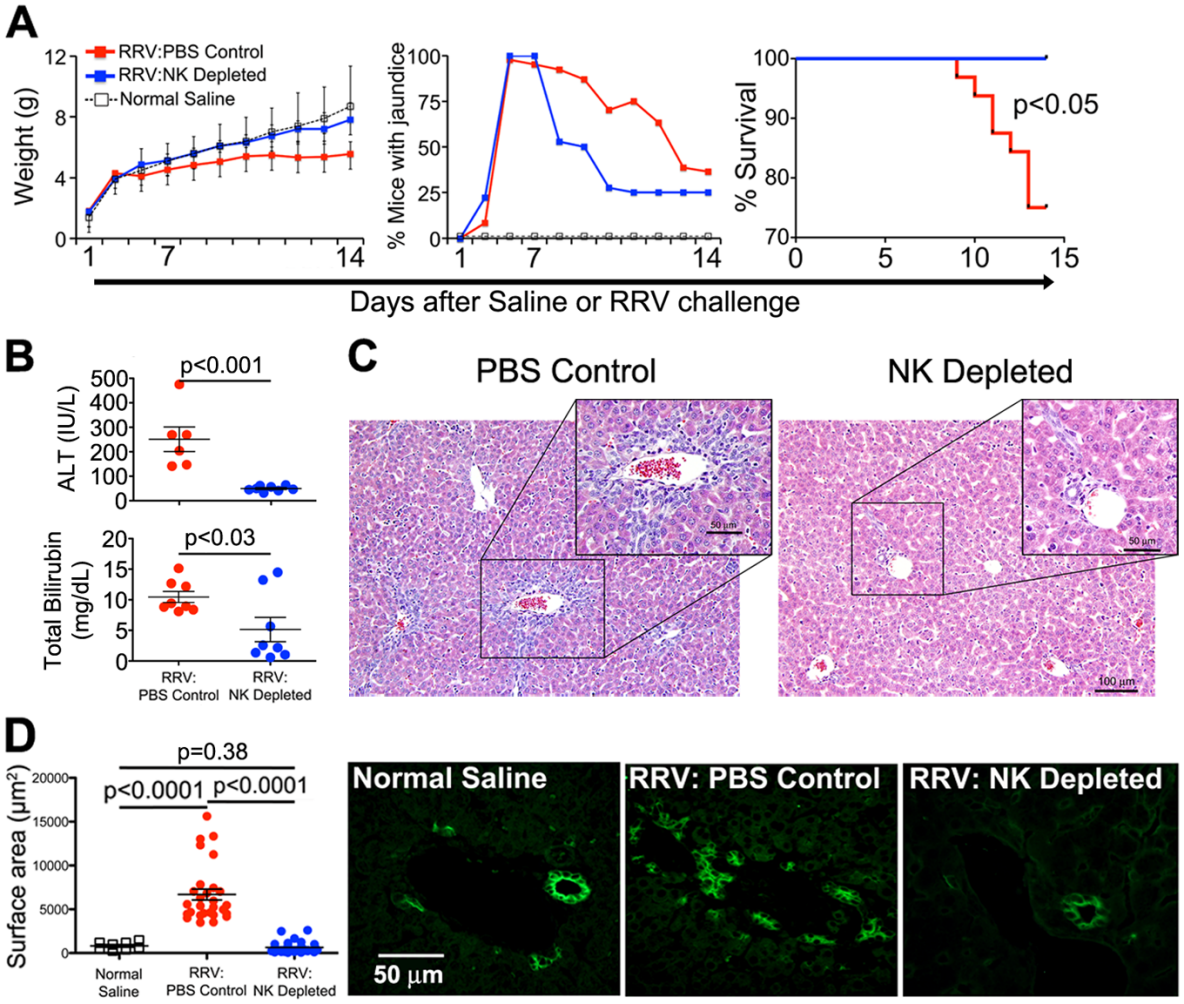
Improved outcome after NK cell depletion in low-dose RRV mice. Depletion of NK cells after the development jaundice (day 6) results in improved growth, earlier resolution of phenotypic jaundice and improved mortality (Kaplan-Meier survival analysis, N = 14–30 per group) (A), lower plasma ALT and total bilirubin (B), and decreased portal inflammation/expansion (C). Panel D contains a dot plot representation of the relative surface area (left graph) of cytokeratin-positive stained portal profiles (representative immunostaining panels on the right) showing a decrease in cholangiocyte profiles in mice undergoing NK cell depletion where each dot represents an individual portal triad (cholangiocytes; green; N = 3–4 mice per group).

**Fig 4:**
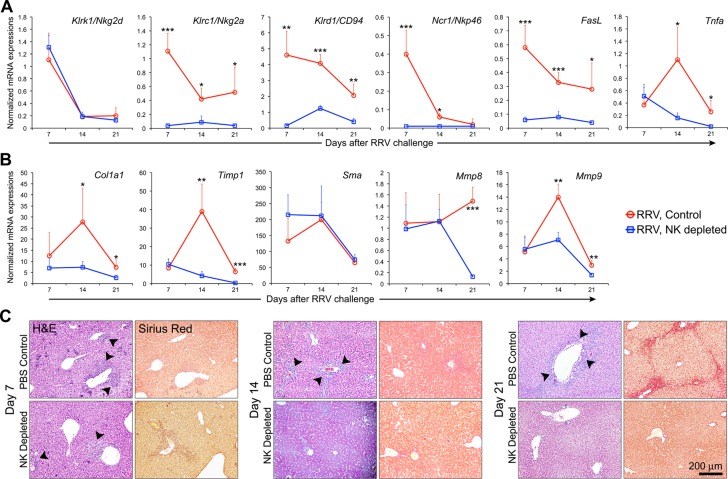
Gene expression, histology and fibrosis after NK cell depletion. Depletion of NK cells after the development of jaundice (day 6) results in significantly decreased hepatic markers of (A) NK cell cytotoxicity and inflammation (*Klrk1/Nkg2d*, *Klrc1/Nkg2a*, *Klrd1/CD94*, *Ncr1/Nkp46*, *FasL*, and *Tnfa*) and (B) fibrosis (*Col1a1*, *Timp1*, *Sma*, *Mmp8*, and *Mmp9*) on days 7, 14 and 21 following low-dose injection. * = P<0.05, ** = P<0.001 and *** = P≤0.0001 (A and B). Panel C contains corresponding liver histology demonstrating persistent periportal expansion and inflammation (arrow heads on H&E) and the development of fibrosis (Sirius Red) on days 7, 14 and 21 that are suppressed by the depletion of NK cells.

The administration of low-dose RRV resulted in the development of jaundice by day of life 6 in all mice; however, notable differences were seen over the subsequent observation period between the groups of mice undergoing NK depletion or PBS control. NK depletion was associated with improved growth, a more rapid resolution of jaundice and increased survival compared to mice receiving PBS (Fig 3A). Biochemical evidence of hepatic injury and biliary obstruction, assessed by plasma ALT and bilirubin, were significantly decreased in NK-depleted mice (Fig 3B and S3 Fig). Microscopic and immunofluorescence analysis of the liver revealed typical portal inflammation and expansion, with increased populations of cholangiocytes by day of life 14 in RRV-infected mice receiving PBS, which was largely prevented by the administration of NK antiserum (Fig 3C and 3D). To determine the effect of NK cell depletion on the progression of the liver injury, we quantified hepatic markers of inflammation and fibrosis and correlated with histological analyses. Overall, NK cell depletion resulted in decreased expression of NK cell cytotoxicity and inflammation (*Klrc1/Nkg2a*, *Klrd1/CD94*, *Ncr1/Nkp46*, *FasL*, and *Tnfa*; expression of *Klrk1/Nkg2d* were not affected) and fibrosis markers (*Col1a1*, *Timp1*, *Mmp8*, and *Mmp9; Sma* expression did not change) (Fig 4A and 4B); there was a notable trend toward lower levels of IL-6, with significant decreases in the concentration of Tnfa and IL-1b at 21 days (S4 Fig). The decreased expression of genes and proteins correlated with improvement in histological features of portal inflammation and fibrosis (Fig 4C; a comprehensive panel including Masson’s Trichrome staining is shown in S2 Fig). Collectively, these data demonstrate that NK cells play an important role in the progression of the intrahepatic hepatobiliary injury of experimental biliary atresia.

### Expression and biological relatedness of extrahepatic genes on day 14 following RRV challenge

**Fig 5:**
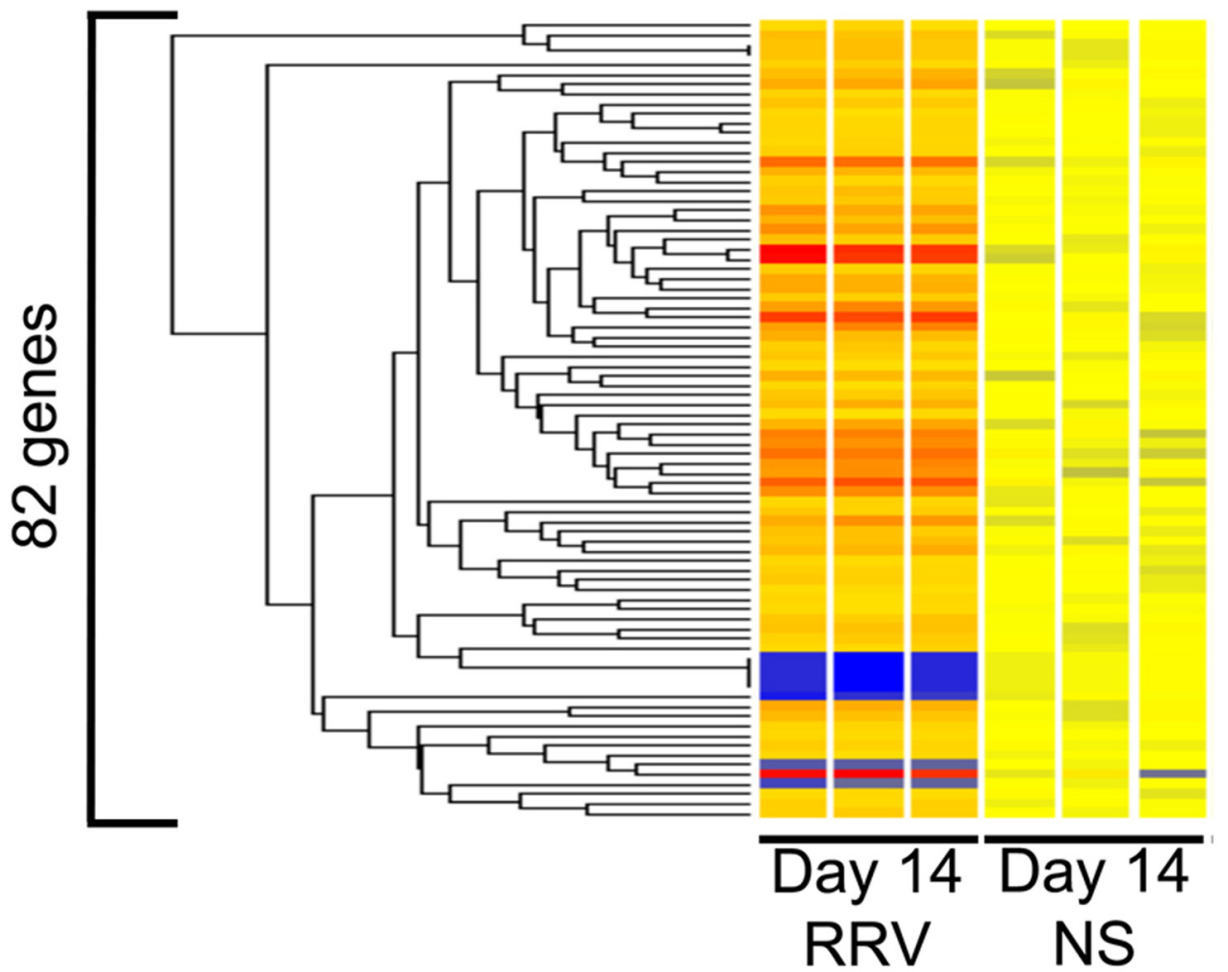
Molecular signature of extrahepatic bile ducts. Condition tree of the 82 genes, showing the level of expression 14 days after RRV or normal saline (NS) control. Levels of expression are depicted as a range in color, in which red represents up-regulation, blue represents down-regulation, and yellow represents baseline levels.

**Fig 6:**
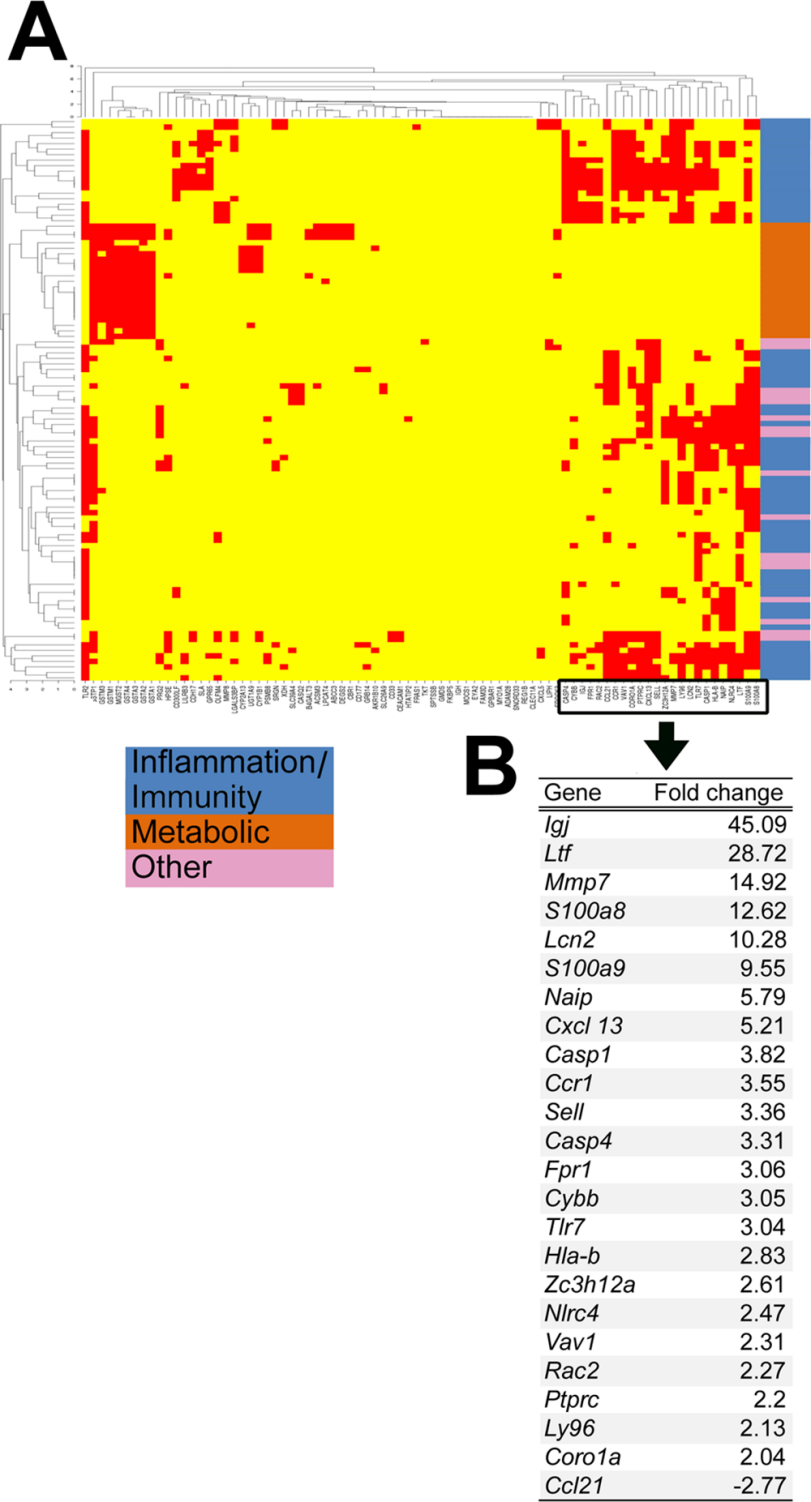
Expression and biological relatedness of genes differentially expressed in extrahepatic bile ducts on day 14 following RRV challenge. (A) A functional enrichment analysis by CIMminer classifies 82 genes into 3 functional categories: inflammation/immunity (blue), metabolic [24] and other (pink). The red areas in the heatmap indicate closely related biological processes that are shared by the subgroup of genes shown on the horizontal axis. (B) Ranking of the top 24 inflammation/immunity based on fold change compared to normal saline controls.

To search for potential mechanisms that mediate the decrease in liver injury induced by NK cell depletion, we quantified the expression of liver genes in NK cell-depleted and control groups. First, based on the lack of published data linking genes or gene groups to the intrahepatic disease in experimental biliary atresia, we analyzed the genome-wide expression data for EHBDs harvested 14 days after injection of full-dose RRV or saline controls published by our group recently [22]. We identified 133 probe sets from a total of 35,557 that were differentially expressed by at least 2 fold (S2 Table). Functional analysis of these probe sets by ToppFun identified 82 known genes that were over- or under-expressed in RRV-injected mice when compared to controls (Fig 5). Interestingly, the assessment of the biological relatedness of the 82 genes showed enrichment for inflammation/immunity and metabolism (Fig 6A) [22].

### Effect of NK cell depletion on intrahepatic gene expression

**Fig 7:**
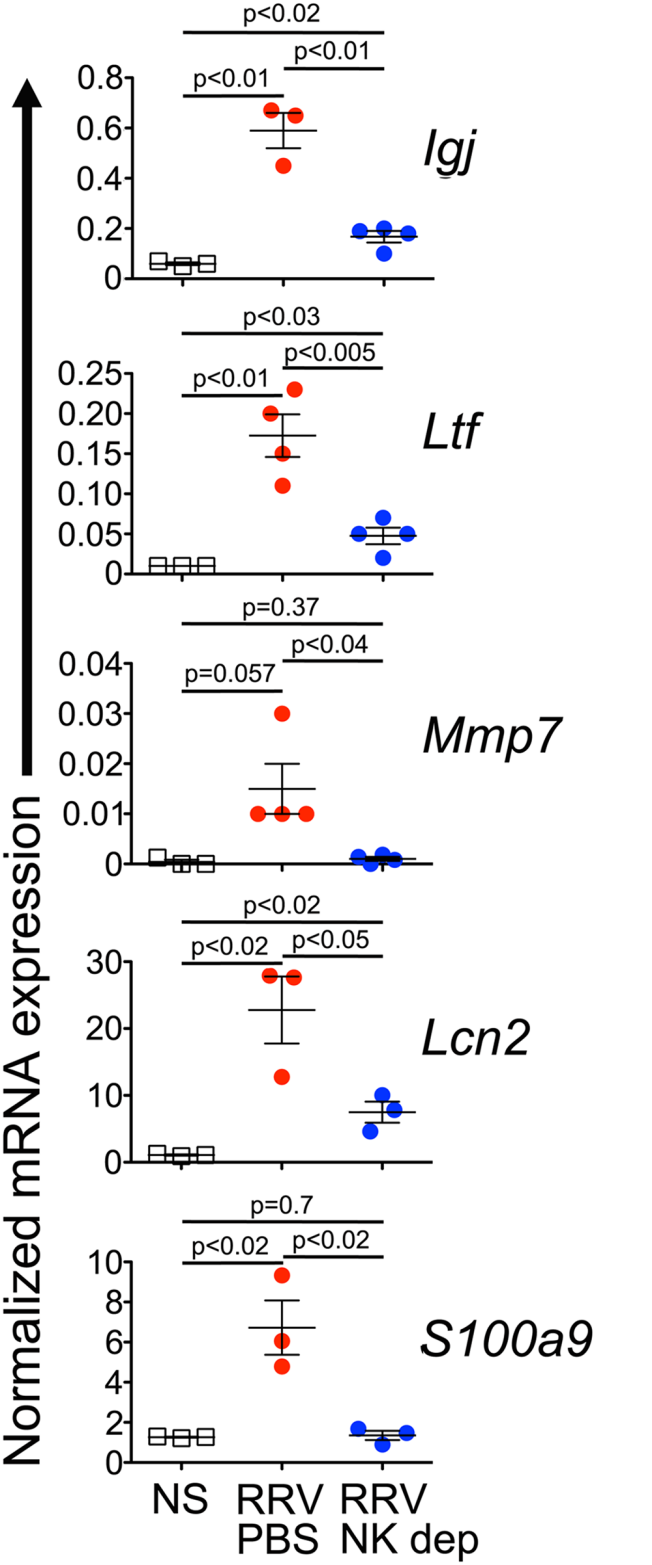
Suppression of hepatic gene expression by NK cell depletion. Decreased mRNA expression of *Igj*, *Ltf*, *Mmp7*, *LCcn2* and *S100a9* following intraperitoneal injections of 30 μL of rabbit anti-asialo GM1 antiserum beginning on day 6 following low-dose RRV infection (N = 3–4 per group).

Based on the role of inflammation/immunity in the pathogenesis of biliary atresia, we ranked the genes based on the levels of expression (Fig 6B), and developed primers to quantify hepatic mRNA expression for the top 6 genes (*Igj*, *Ltf*, *Mmp7*, *Lcn2*, *S100a8* and *S100a9*); the levels of expression of these genes increased 9.55–45.09 fold over controls. The pattern of expression in the liver was similar to the microarray data for extrahepatic bile ducts, increasing for *Igj*, *Ltf*, *Mmp7*, *Lcn2*, and *S100a9* by 6–247 fold above controls (S5 Fig); the fold change for *S100a8* was not reproduced. To directly investigate whether the expression of these genes was linked to the hepatic expansion of the NK cell population after low-dose RRV, we quantified their mRNA expression in NK cell-depleted and control livers 14 days after low-dose RRV challenge. NK cell depletion significantly suppressed the expression of *Igj*, *Ltf*, *Mmp7*, *Lcn2* and *S100a9* (Fig 7).

## Discussion

Our findings point to an integral role of NK cells in the development of the inflammatory and fibrosing liver disease induced by RRV infection in experimental biliary atresia, and demonstrate that the depletion of these cells at the onset of jaundice and biliary obstruction can improve cholestasis, decrease tissue inflammation, prevent the development of fibrosis, and improve survival. While the high mortality by 2 weeks of age in newborn mice receiving the traditional high dose of RRV has not been suitable for studies of the liver injury in experimental atresia, the use of a lower viral load overcame this investigational challenge by allowing better survival while inducing a persistent, progressive inflammatory liver injury. Capitalizing on these findings, we directly explored whether the effector role of NK cells demonstrated in the initiation of epithelial injury of extrahepatic bile ducts [13] extended to the intrahepatic hepatobiliary injury. In keeping with the anatomical continuity along the biliary tract, depletion of NK cells decreased the liver inflammation that was typical of surviving mice.

The injury and obstruction of extrahepatic bile ducts induced by the smaller inoculum of RRV share morphological features of high-dose RRV; however, the epithelial repair and recanalization of the duct lumen by 14 days suggest that the severity of the injury is proportional to the viral load. Despite the milder phenotype in extrahepatic bile ducts, the extension of the biliary injury into the intrahepatic environment persisted and the type-1 response in the liver was qualitatively and quantitatively similar to the full-dose RRV, as demonstrated by the expression of *Ifnγ*, *Cxcl9* and *Cxcl10*. Low-dose RRV also induced a prominent expansion of CD8+ T- and NK lymphocytes. Collectively, these data show that the severity of experimental extrahepatic atresia relates to the magnitude of the infection, with an intrahepatic biliary injury that is present in all doses and progresses at a lower dose.

By modifying the infectious model and beginning the depletion of NK cells after the onset of jaundice in neonatal mice, we aimed at replicating the clinical scenario where icteric infants already have complete obstruction of the EHBD at the time of a biliary atresia diagnosis. This differs from previous reports that focused on the key initiators of cholangiocyte injury through the depletion of individual cell population or blocking of cytokines before or shortly after RRV infection [4, 6, 13]. Seeking to understand the molecular circuits that are activated beyond the time of duct obstruction, we found high expression of genes related to inflammation/immunity (*Igj*, *Ltf*, *Lcn2* and *S100a9*) and tissue repair (*Mmp7*) in EHBDs and livers. In this setting, the depletion of NK cells efficiently decreased the expansion of effector mononuclear cells and suppressed the expression of these genes. These findings combined with the decrease in liver inflammation, suppressed bilirubin levels, lower intrahepatic markers of fibrosis and improved survival suggest that NK cells play a key regulatory role in the liver disease of experimental biliary atresia.

Mechanistic studies in the RRV model of biliary atresia have advanced the understanding of initiation and progression of epithelial injury and obstruction of EHBDs. Among the limitations of the model as it reflects mechanisms relevant to the human disease [14] is the inability to analyze the mechanisms of liver injury. Here, we sought to overcome this limitation by using a lower inoculum of RRV to induce tissue injury that largely recapitulates the high-dose model in histology, infiltration of effector lymphocytes and gene expression, but allowed for the development of progressive intrahepatic disease. Future follow-up studies will determine if other mediators that have been shown to regulate extrahepatic injury, including but not limited to CD8+ T-lymphocytes [6], IFNγ [4], B cells [25] and perforin and granzyme [5], are also important for the intrahepatic injury in mice that survive the initial duct obstruction.

In summary, the administration of a lower dose of RRV induced an obstructive extrahepatic cholangiopathy while allowing for restoration of the duct epithelium and the development of a progressive intrahepatic hepatobiliary injury that shares the molecular and cellular signatures of the full dose model. Exploring the mechanisms of liver injury, we found that NK cell depletion lowered plasma ALT and bilirubin levels, and reduced hepatic inflammation and fibrosis. The choice of starting NK cell depletion after the onset of jaundice aimed at recapitulating the time of diagnosis of biliary atresia in humans, thus raising the possibility that depletion of NK cells may constitute a therapeutic strategy to block progression of disease and improve the outcome of children with this devastating disease.

## Supporting Information

S1 Fig Luminal obstruction of bile ducts after low-dose RRV. Sections of mouse extrahepatic bile ducts (EHBDs) with the corresponding livers 7 days after low-dose RRV infection on day 1 of life. EHBDs have luminal obstruction by inflammatory cells and livers have expansion of portal tracts by inflammatory cells. Tissue sections were stained with H&E
(TIF)

S2 Fig Hepatic inflammation and fibrosis after NK cell depletion in low-dose RRV mice. Liver sections show persistent periportal inflammatory infiltrate from days 7–21, with the development of fibrosis on day 21 after 0.25x10^6^ ffu (low-dose) RRV inoculation. Inflammatory and fibrotic changes are ameliorated by NK cell depletion. Tissue sections stained with H&E, Sirius Red and Massons Trichrome (where noted); liver magnification x20; N = 4–11 per group at each time point.
(TIF)

S3 Fig ALT and total bilirubin levels after NK cell depletion in low-dose RRV mice. Plasma ALT (A) and total bilirubin (B) is significantly decreased on days 14 and 21 in NK-depleted mice compared to controls after receiving NK-depleting antibody.
(TIF)

S4 Fig Plasma cytokine levels in control and NK-depleted mice after RRV. Depletion of NK cells after the development of jaundice (day 6) resulted in significantly decreased levels of circulating Tnfα, IL1β and IL6 at 12 days after low-dose RRV. N = 4–8 per group; ** = P<0.001.
(TIF)

S5 Fig Comparison of gene expression in livers and extrahepatic bile ducts (EHBDs) after RRV. mRNA expression by microarray (for EHBDs) and qPCR (for livers) 14 days after high-dose RRV on day 1 of life. mRNA is depicted as fold change over saline controls.
(TIF)

S1 Table Forward (For) and reverse (Rev) oligonucleotide primer sequences and annealing temperatures (Tm) used in real-time PCR to quantify the expression of cytokines, chemokines and NK-cell enriched genes. (DOCX)

S2 Table 133 probe sets with accompanying genes identified from a total of 35,557 sets that were differentially expressed by at least 2 fold in the extrahepatic bile ducts of high-dose infected mice at day 14 following RRV challenge compared to normal saline controls. (DOCX)
