# Supplementary figures and images for: Natural Killer Cells Promote Long-Term Hepatobiliary Inflammation in a Low-Dose Rotavirus Model of Experimental Biliary Atresia
Source: PLoS One. 2015 May 19;10(5):e0127191. doi: 10.1371/journal.pone.0127191 (PMC4437784; doi:10.1371/journal.pone.0127191)

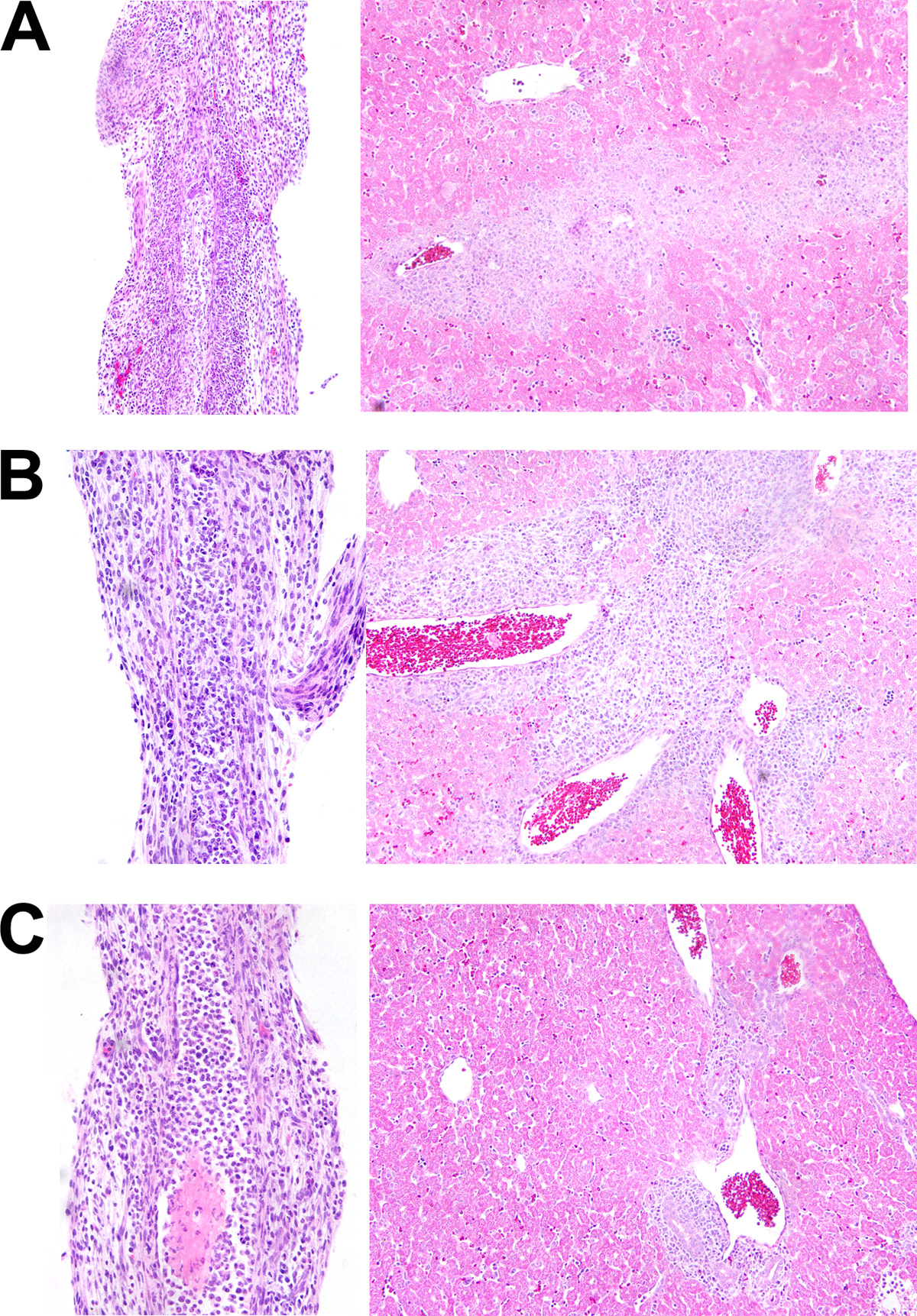

Supplement: S1 Fig — Sections of mouse extrahepatic bile ducts (EHBDs) with the corresponding livers 7 days after low-dose RRV infection on day 1 of life. EHBDs have luminal obstruction by inflammatory cells and livers have expansion of portal tracts by inflammatory cells. Tissue sections were stained with H&E (TIF) [file pone.0127191.s001.tif]

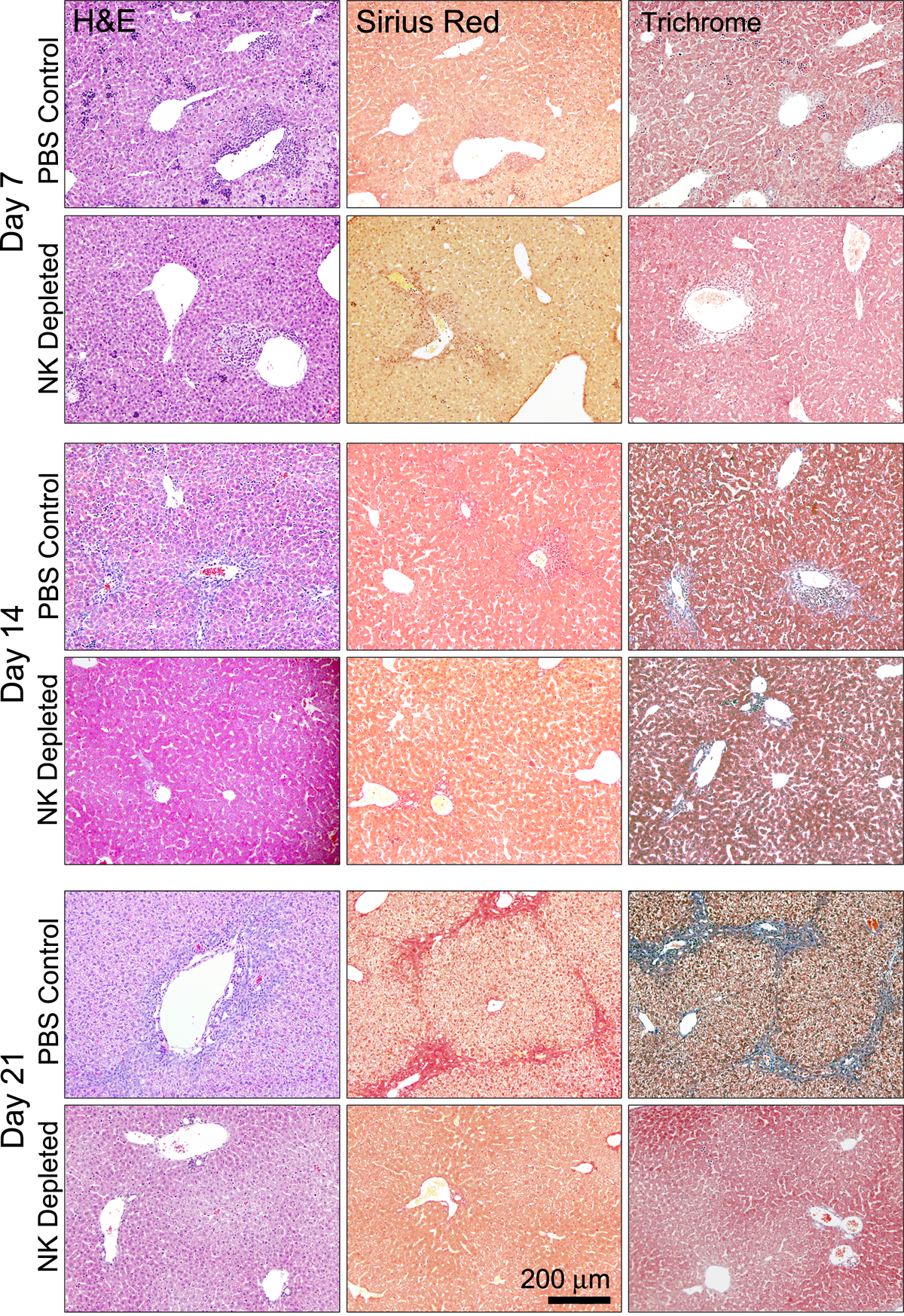

Supplement: S2 Fig — Liver sections show persistent periportal inflammatory infiltrate from days 7–21, with the development of fibrosis on day 21 after 0.25x10^6^ ffu (low-dose) RRV inoculation. Inflammatory and fibrotic changes are ameliorated by NK cell depletion. Tissue sections stained with H&E, Sirius Red and Massons Trichrome (where noted); liver magnification x20; N = 4–11 per group at each time point. (TIF) [file pone.0127191.s002.tif]

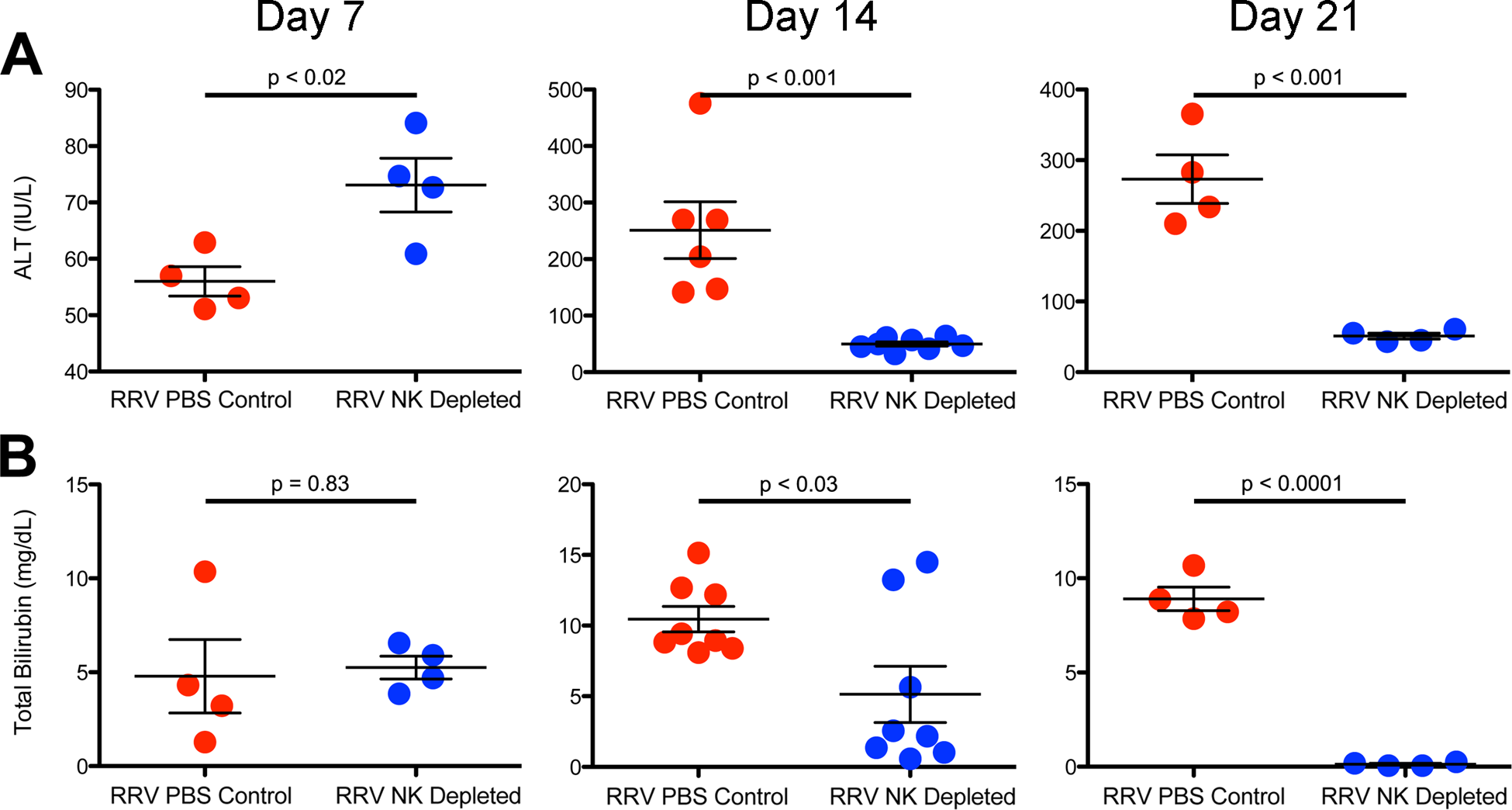

Supplement: S3 Fig — Plasma ALT (A) and total bilirubin (B) is significantly decreased on days 14 and 21 in NK-depleted mice compared to controls after receiving NK-depleting antibody. (TIF) [file pone.0127191.s003.tif]

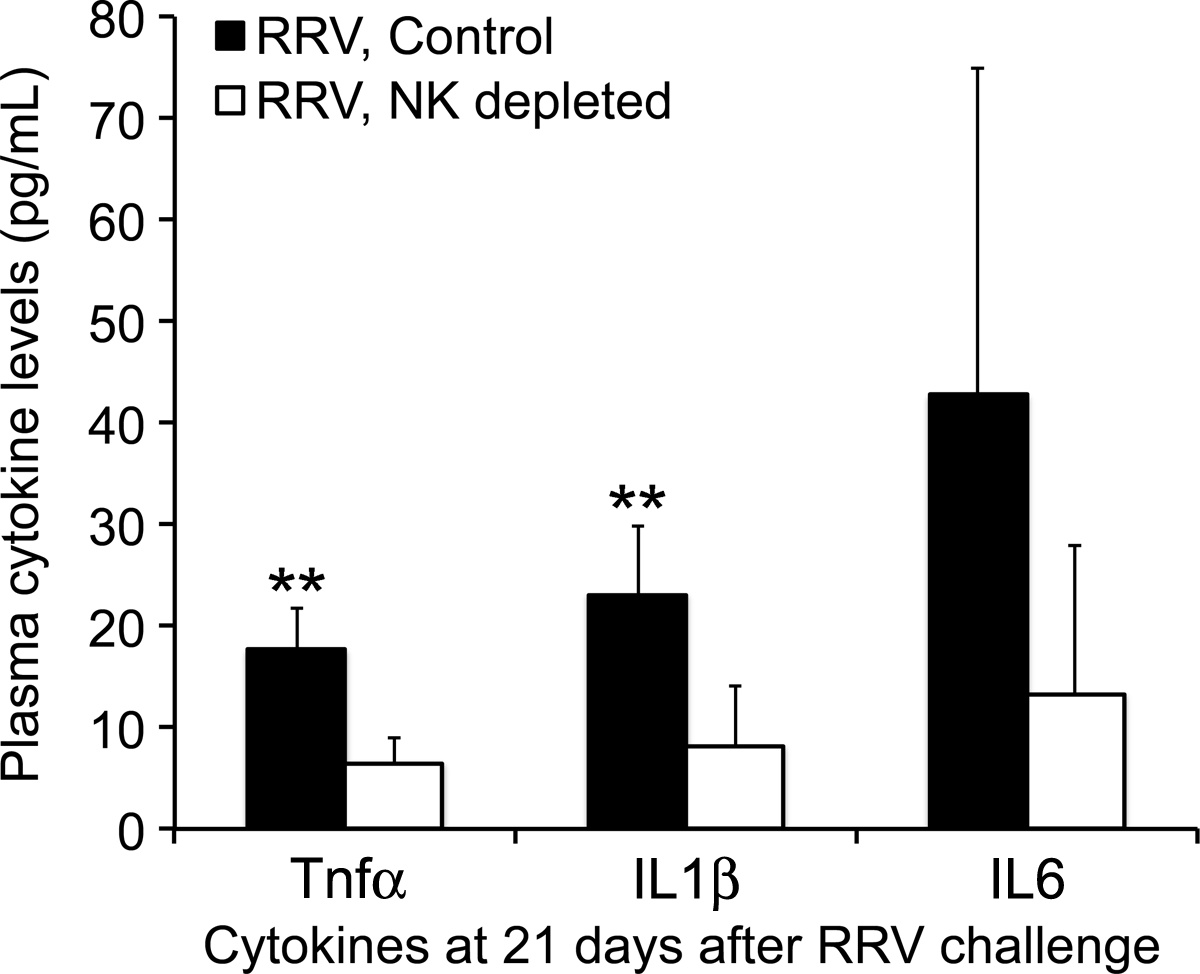

Supplement: S4 Fig — Depletion of NK cells after the development of jaundice (day 6) resulted in significantly decreased levels of circulating Tnfα, IL1β and IL6 at 12 days after low-dose RRV. N = 4–8 per group; ** = P<0.001. (TIF) [file pone.0127191.s004.tif]

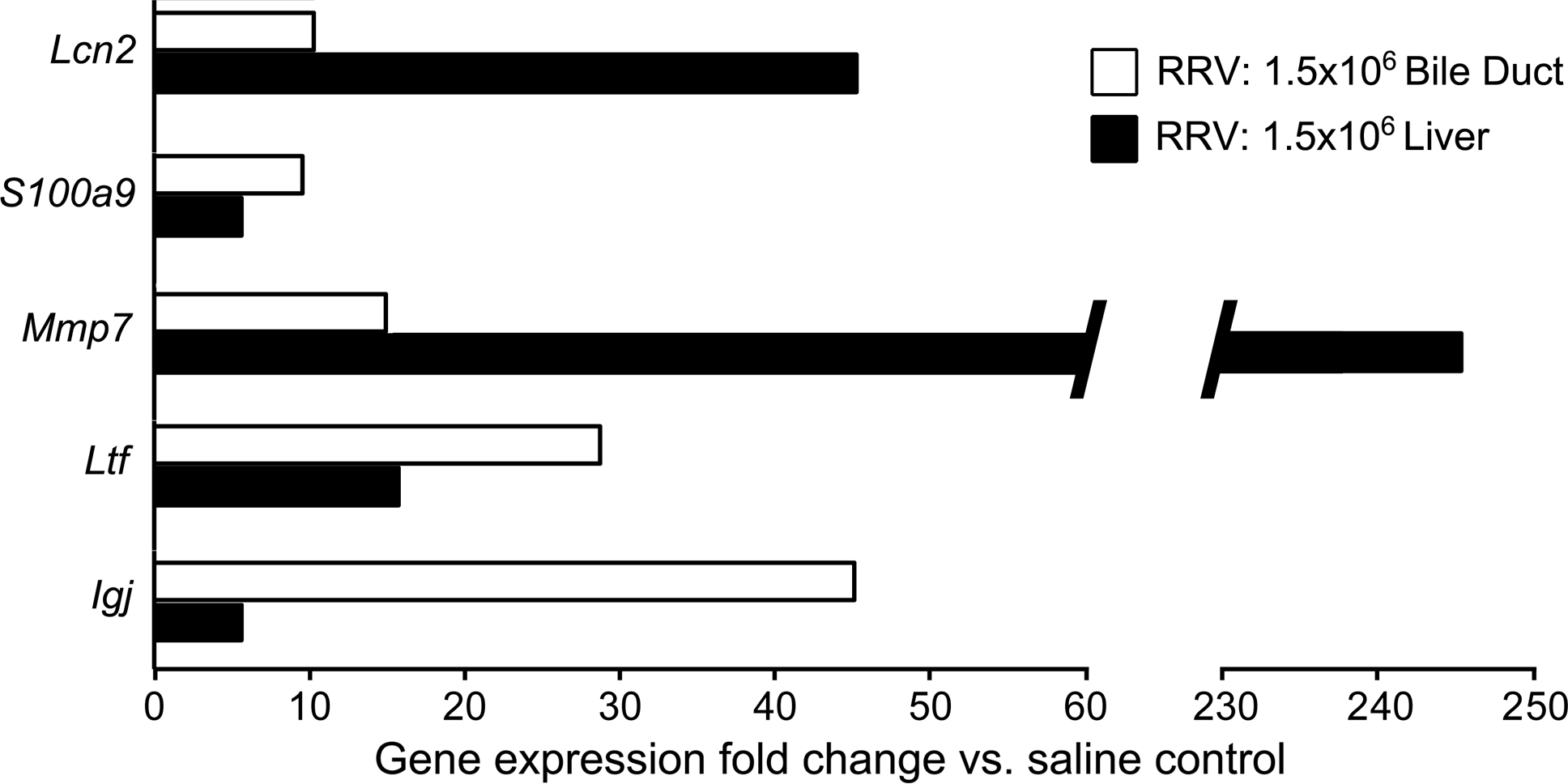

Supplement: S5 Fig — mRNA expression by microarray (for EHBDs) and qPCR (for livers) 14 days after high-dose RRV on day 1 of life. mRNA is depicted as fold change over saline controls. (TIF) [file pone.0127191.s005.tif]
