# Supplementary material for: Natural Killer Cells Promote Long-Term Hepatobiliary Inflammation in a Low-Dose Rotavirus Model of Experimental Biliary Atresia
Source: PLoS One. 2015 May 19;10(5):e0127191. doi: 10.1371/journal.pone.0127191 (PMC4437784; doi:10.1371/journal.pone.0127191)
Supplement: S1 Table — (DOCX) [file pone.0127191.s006.docx]

**Supplemental Table 1.** Forward (For) and reverse (Rev) oligonucleotide primer sequences and annealing temperatures (Tm) used in real-time PCR to quantify the expression of cytokines, chemokines and NK-cell enriched genes

| *Name* | Sequence 5' to 3' |  | Tm |
| --- | --- | --- | --- |
| *Ifng* | For: GGCTGTCCCTGAAAGAAAGC |  | 52 |
|  | Rev: GAGCGAGTTATTTGTCATTCGG |  | 52 |
| *Cxcl9* | For: GAGCTAGATAGACCTCACCAAG |  | 52 |
|  | Rev: CCATTAGCACCATCTCTGA |  | 52 |
| *Cxcl10* | For: TCGCTCAAGTGGCTGGGATG |  | 57 |
|  | Rev: TAGGGAGGACAAGGAGGGTGTG |  | 57 |
| *Igj* | For: ACCCTTTCCTTCCTTCCTTTG |  | 60.6 |
|  | Rev: AACTTCCTGTGTTCATTGGCTG |  | 60.8 |
| *Ltf* | For: CAAAAGGATAGATTCCCCAACC |  | 60.8 |
|  | Rev: AGACACCTCAAGGCTCCAGC |  | 64.5 |
| *Mmp7* | For: GCACATCAGTGGGAACAGGC |  | 64.5 |
|  | Rev: GCATTTCCTTGAGGTTGTCCAC |  | 62.7 |
| *S100a8* | For: ACTTCGAGGAGTTCCTTGCG |  | 62.4 |
|  | Rev: TCTGTAGACATATCCAGGGACCC |  | 64.6 |
| *Lcn2* | For: TTCACCCGCTTTGCCAAG |  | 59.9 |
|  | Rev: GCCACACTCACCACCCATTC |  | 64.5 |
| *S100a9* | For: CAGCATAACCACCATCATCGAC |  | 62.7 |
|  | Rev: TCATAAAGGTTGCCAACTGTGC |  | 60.8 |
| *Mmp8* | For: ATCCTTGCCCATGCCTTTCA |  | 60.4 |
|  | Rev: TTCATGAGCAGCCACGAGAA |  | 60.4 |
| *Mmp9* | For: TACAGGGCCCCTTCCTTACT |  | 62.4 |
|  | Rev: ACACCCACATTTGACGTCCA |  | 60.4 |
| *Col1a1* | For: TGGTGCTAAGGGTGAAGCTG |  | 62.4 |
|  | Rev: TCCATCAGCACCAGGGTTTC |  | 62.4 |
| *Sma* | For: TCACCAACTGGGACGACATG |  | 62.4 |
|  | Rev: ATTTTCTCCCGGTTGGCCTT |  | 60.4 |
| *Timp1* | For: TTCTTGGTTCCCTGGCGTAC |  | 62.4 |
|  | Rev: ACTCTCCAGTTTGCAAGGGA |  | 60.4 |
| *Ncr1* | For: ATGGGAACATCCAAGCAGAG |  | 60.4 |
|  | Rev: CAGGCTCACTGGGAAAAGAC |  | 62.4 |
| *Klrk1* | For: GGCTTGCCATTTTCAAAGAG |  | 58.4 |
|  | Rev: ATCCAGTTGTTAGGGCATGG |  | 60.4 |
| *Klrd1* | For: TCGGTGGAGACTGATGTCTG |  | 62.4 |
|  | Rev: AGTGGTGGTTGGAGAAGGTG |  | 62.4 |
| *Klrc1* | For: TGCAAAGGTTTTCCATGTCC |  | 58.4 |
|  | Rev: GCTTCGGTATATGGTGTGGC |  | 62.4 |
| *FasL* | For: GCAGAAGGAACTGGCAGAAC |  | 62.4 |
|  | Rev: TTAAATGGGCCACACTCCTC |  | 60.4 |
| *Tnfa* | For: AAGGGAGAGTGGTCAGTTGCC |  | 54 |
|  | Rev: CCTCAGGGAAGAGTCTGGAAAGG |  | 54 |
