# Supplementary material for: Natural Killer Cells Promote Long-Term Hepatobiliary Inflammation in a Low-Dose Rotavirus Model of Experimental Biliary Atresia
Source: PLoS One. 2015 May 19;10(5):e0127191. doi: 10.1371/journal.pone.0127191 (PMC4437784; doi:10.1371/journal.pone.0127191)
Supplement: S2 Table — (DOCX) [file pone.0127191.s007.docx]

**Supplemental Table 2.** 133 probe sets with accompanying genes identified from a total of 35,557 sets that were differentially expressed by at least 2 fold in the extrahepatic bile ducts of high-dose infected mice at day 14 following RRV challenge compared to normal saline controls.

| Probe set ID | Gene Symbol | Fold change  (Day 14-RRV vs Day 14-Saline) |
| --- | --- | --- |
| 10339469 | --- | -2.5732217 |
| 10344966 | Ly96 | 2.1257544 |
| 10345065 | Gsta3 | 4.269098 |
| 10347291 | --- | 5.0392466 |
| 10347307 | Gpbar1 | -2.0620432 |
| 10348354 | Ugt1a9 | 2.0215418 |
| 10349671 | Slc26a9 | 4.395377 |
| 10351197 | Sell | 3.3618934 |
| 10351563 | B4galt3 | 2.4251401 |
| 10358224 | Ptprc | 2.1994588 |
| 10360377 | --- | 3.8455129 |
| 10360406 | Ifi205 | 5.5735598 |
| 10361152 | Gstp2 | 2.296776 |
| 10362511 | LOC667824 | 2.140734 |
| 10366994 | Myo1a | 3.4100523 |
| 10368477 | --- | 2.3196926 |
| 10369615 | Srgn | 3.1930912 |
| 10376885 | --- | 2.1050196 |
| 10379630 | Slfn2 | 2.051341 |
| 10382844 | --- | 3.2653964 |
| 10385518 | Tgtp | 2.7124848 |
| 10385533 | Tgtp | 2.7280982 |
| 10386093 | --- | 3.2653964 |
| 10386197 | --- | 7.7621684 |
| 10389894 | Abcc3 | 4.826089 |
| 10392845 | Cd300lf | 3.3148172 |
| 10393573 | Lgals3bp | 2.0617611 |
| 10397645 | Gpr65 | 2.509139 |
| 10402575 | Degs2 | 2.1321514 |
| 10402864 | Igh-6 | 2.4963999 |
| 10403028 | Igh // Igh | 2.7081177 |
| 10403034 | --- | 4.1346455 |
| 10403048 | Igh // Igh // Igh | 3.0175445 |
| 10403054 | Igh-VJ558 // Igh-VJ558 // Igh-VJ558  // Igh-VJ558 // Igh-VJ558 | 3.1474972 |
| 10408531 | Gmds | 2.4572065 |
| 10411611 | Naip5 | 5.7940702 |
| 10413542 | Tkt | 2.3158162 |
| 10415438 | Mcpt2 | 10.064523 |
| 10416689 | Olfm4 | 7.0891824 |
| 10417568 | Oit1 | 3.1582763 |
| 10421143 | Adam28 | 2.6108499 |
| 10424683 | --- | 4.506501 |
| 10429128 | Sla | 2.263613 |
| 10429568 | Ly6c1 | 3.8032575 |
| 10429573 | Ly6c2 | 3.3128488 |
| 10430006 | Slc39a4 | 2.5836425 |
| 10430372 | Rac2 | 2.266917 |
| 10432640 | --- | 2.1203463 |
| 10435497 | Stfa2 | 29.059195 |
| 10435501 | Stfa1 | 2.2936375 |
| 10436967 | Cbr1 | 2.9962225 |
| 10438405 | Igl-V1 | 8.248696 |
| 10438592 | Liph | 2.2893128 |
| 10439292 | BC100530 | 9.428824 |
| 10439296 | Stfa2 | 9.496066 |
| 10439299 | Stfa3 | 8.730121 |
| 10444258 | Psmb8 | 2.6985106 |
| 10444780 | H2-D1 | 2.8331587 |
| 10444814 | --- | 3.064482 |
| 10444821 | H2-Q8 | 2.9818416 |
| 10444824 | H2-Q6 | 2.9363353 |
| 10444830 | H2-Q7 | 2.1832137 |
| 10445826 | Mocs1 | 2.1409614 |
| 10446253 | Vav1 | 2.308056 |
| 10448124 | Fpr1 | 3.0599692 |
| 10449452 | Fkbp5 | 5.631511 |
| 10450699 | --- | 2.0791223 |
| 10450733 | --- | 2.1083703 |
| 10452815 | Xdh | 2.6564705 |
| 10452879 | Nlrc4 | 2.4749532 |
| 10453057 | Cyp1b1 | 3.203542 |
| 10464137 | --- | 2.3072305 |
| 10464583 | Gstp1 | 2.0464504 |
| 10464586 | Gstp2 | 2.2841349 |
| 10473399 | Prg2 | 2.3974774 |
| 10474526 | Agpat7 | 2.0080566 |
| 10478698 | Eya2 | 2.4287639 |
| 10481627 | Lcn2 | 10.2796955 |
| 10483163 | Grb14 | -2.0827706 |
| 10491952 | Mgst2 | 2.8617547 |
| 10493831 | S100a8 | 12.61604 |
| 10494804 | Casq2 | -3.8629286 |
| 10498653 | 1110032A04Rik | 2.706728 |
| 10498992 | Tlr2 | 3.1895108 |
| 10499861 | S100a9 | 9.548228 |
| 10501218 | Gstm3 | 2.9511893 |
| 10501229 | Gstm1 | 2.0587387 |
| 10503341 | Cdh17 | 4.0130906 |
| 10504127 | Ccl21a | -2.76608 |
| 10504154 | Ccl21c | -2.76608 |
| 10504183 | Ccl21b | -2.76608 |
| 10512377 | Ccl21c | -2.76608 |
| 10516266 | Zc3h12a | 2.6107934 |
| 10523120 | Cxcl5 | 6.7086334 |
| 10523359 | Cxcl13 | 5.20504 |
| 10523376 | Fras1 | -2.5938458 |
| 10531126 | Igj | 45.09388 |
| 10531737 | Hpse | 2.091907 |
| 10537146 | Akr1b8 | 6.4382534 |
| 10538903 | Igk-V21-4 | 7.738517 |
| 10539194 | Reg2 | 118.17483 |
| 10541564 | Clec4a3 | 2.030691 |
| 10551226 | Cyp2a4 | 5.101574 |
| 10553403 | Htatip2 | 2.1184986 |
| 10556769 | Acsm3 | 4.386147 |
| 10559467 | Pira6 | 2.8480866 |
| 10560886 | Cd177 | 2.0457423 |
| 10561008 | Ceacam1 | 4.02849 |
| 10562709 | Cd33 | 2.225861 |
| 10562761 | Clec11a | -2.4325988 |
| 10563112 | Snord33 | 3.4253504 |
| 10568024 | Coro1a | 2.0356321 |
| 10569020 | Ifitm6 | 9.094833 |
| 10572024 | Spock3 | -2.083981 |
| 10574149 | --- | 3.3024282 |
| 10580663 | AU018778 | 18.429026 |
| 10582985 | Casp1 | 3.8158417 |
| 10582997 | Casp4 | 3.3145967 |
| 10583100 | Mmp8 | 5.432882 |
| 10583133 | Mmp7 | 14.918681 |
| 10586168 | --- | 3.961208 |
| 10587315 | Gsta4 | 3.9120197 |
| 10587323 | Gsta2 | 84.81259 |
| 10587331 | Gsta1 | 71.295815 |
| 10589535 | Ngp | 16.35463 |
| 10589703 | Ltf | 28.71863 |
| 10595145 | EG666383 | 2.8232157 |
| 10598004 | Ccr1 | 3.549497 |
| 10598023 | --- | 3.3556626 |
| 10598027 | --- | 2.7128832 |
| 10603551 | Cybb | 3.0535772 |
| 10607870 | Tlr7 | 3.035904 |
| 10608637 | --- | 2.9879386 |
|  |  |  |
